# MicroRNA expression profile in Treg cells in the course of primary immune thrombocytopenia

**DOI:** 10.1136/jim-2019-001020

**Published:** 2019-07-03

**Authors:** Yuandong Zhu, Huan Zhu, Xiaobao Xie, Zhuojun Zheng, Yun Ling

**Affiliations:** Department of Hematology, The Third Affiliated Hospital, Soochow University, Changzhou, China

**Keywords:** blood platelets, microarray analysis

## Abstract

Primary immune thrombocytopenia (ITP) is an autoimmune bleeding disorder which characterizes with platelet production impairment and platelet destruction increment. CD4^+^CD25^+^ *Foxp*3^+^ Treg cells (Tregs) are involved in the immune pathogenesis of ITP. MicroRNAs (miRNAs) are also involved in ITP and their loss of function is shown to facilitate immune disorders. Thus, the miRNA expression profile in Tregs from ITP was analyzed in this study. We assessed the genome-wide miRNA expression profile of three newly diagnosed adult patients with ITP and three healthy controls using microarray analysis of CD4^+^CD25^+^CD127^dim/−^ Tregs that were sorted using an immune magnetic bead kit. The miRNA microarray chip was based on miRBase 18.0 and Volcano Plot filtering software used to analyze the miRNA profile in Tregs. Distinct miRNA expression was further validated by fluorescence-based real-time quantitative PCR (qPCR). We found that 502 human miRNAs were differentially expressed (244 upregulated and 258 downregulated) in patients with ITP compared with healthy donors. We identified 37 miRNAs expressed significantly, including 26 upregulated and 11 downregulated. Among the deregulated miRNAs, three downregulated miRNAs including miR-155–5p, miR-146b-5p, and miR-142–3p were selected for qPCR verification. We confirmed that miR-155–5p, miR-146b–5p, and miR-142–3p were significantly decreased in Tregs from patients with ITP compared with healthy controls. Compared with the healthy controls, miRNAs expressed differentially in the Tregs of patients with ITP. The levels of expression of miR-155–5p, miR-146b-5p, and miR-142–3p were significantly decreased. Therefore, the deregulation of miRNAs may affect the function of Tregs in the course of ITP.

**Significance of this study**

**What is already known about this subject?**

- Treg cells have been involved are in the immune pathogenesis of immune thrombocytopenia (ITP). Loss of function of microRNAs is shown to facilitate immune disorders. The miRNA expression profile in Tregs remains unclear.

**What are the new findings?**

- miR-155–5p, miR-146b–5p, and miR-142–3p were significantly decreased in Tregs from patients with ITP compared with healthy.

**How might these results change the focus of research or clinical practice?**

- The results may provide new implication for novel therapeutic approaches for ITP.

## Introduction

Primary immune thrombocytopenia (ITP) is an autoimmune disease. A decreased platelet count could be observed in ITP because of autoantibody-mediated platelet destruction and insufficient platelet production.^1^ Massive bleeding is a serious complication in these patients. The pathogenesis of ITP, including the immune, genetic, and environmental factors remains unclear. One pathogenic factor is a regulatory Tregs disorder that results in antiplatelet antibodies generated by B cells.^2^ Tregs are important immune-regulating cells that maintain immune tolerance and play an important role in a variety of autoimmune diseases.^3^ Tregs are a specialized subpopulation of T cells that act to suppress the activation of the immune system and thereby maintain homeostasis and tolerance to self-antigens. Tregs are a CD4^+^ subset, characterized by CD25^high^ expression, because immunoregulatory function is absent in CD4^+^CD25^low/neg^ cells. Moreover, CD4^+^CD25^+high^ T cells are characterized by a high expression of the forkhead/winged-helix transcription factor 3 (*Foxp3*) gene. Patients with ITP have a Treg immunodeficiency, including a reduction in the number of Tregs and/or weakened immunosuppressive function.^4^

MicroRNAs (miRNAs) are short (about 22 nts) noncoding RNAs that control gene expression at the post-transcriptional level and have been proven to play critical roles in the development, differentiation, and function of immune cells. Recently, miRNAs have been shown to play an important role in the immunosuppressive function of Tregs.^5^ Tregs have their own particular miRNA expression profiles. Cobb *et al* reported that the miRNA expression profile in Treg is distinct from those in conventional CD4^+^ T cells.^6^ Moreover, miR-155 inhibition sensitizes CD4^+^ Th cells for Treg-mediated suppression.^7^ We therefore intended to explore the potential correlation of miRNA during development of Treg dysfunction in the course of ITP.

## Materials and methods

### Patients and health controls

There were 21 newly diagnosed adult patients with ITP (16 females and 5 males, mean age 34±12 years) enrolled in this study. The diagnosis of ITP was based on recently reported criteria.^8^ Of the selected patients, their platelet counts ranged from 7 to 25×10^9^/L, and we excluded tumor disease, autoimmune disease, peptic ulcer, severe infection, and pregnancy.

There were 18 healthy volunteers (14 females and 4 males, mean age 35±10 years) included as the control group with platelet counts ranged from 125 to 260×10^9^/L. The differences between two groups regarding to sex and age was not statistically significant.

The miRNA array study was carried out in 3 patients and 3 age-matched and sex-matched healthy donors and then used fluorescence-based real-time quantitative PCR (qPCR) to measure selected miRNAs from the remaining 18 patients and 15 healthy controls. This study was approved by our hospital-based ethics committee and informed consent was obtained from the patients as legal guardians.

### Isolation of peripheral blood mononuclear cells

Peripheral blood was collected into EDTA-anticoagulated vacuum tubes. Peripheral blood mononuclear cells were isolated using lymph preparation density gradient centrifugation (Reagent Factory, Shanghai, China). A microscope was used to count cells.

### Isolation of Tregs using immune magnetic beads

Purification of CD4^+^CD25^+high^CD127^dim/−^ regulatory cells was achieved in a two-step procedure, following the protocols of two Miltenyi Biotec kits for isolation of Tregs. Non-CD4^+^ MACSxpress depletion cocktail and CD25 MicroBeads were used for labeling. In the first step, we used immunomagnetic depletion with MACSxpress Beads to remove the MACSxpress Separator non-CD4^+^ cells and the majority of CD127^hi^ cells after erythrocytes were aggregated and sedimented. The CD25 MicroBeads may not be influenced by the magnetic field of the MACSxpress Separator (Miltenyi Biotec, Germany) because of its small nature. In the second step, anti-CD25-coated microbeads were directly labeled to the CD4^+^CD127^dim/−^ T cells. Then, CD4^+^CD127^dim/−^ T cells in the pre-enriched CD4^+^ T cell fraction were isolated according to positive selection. After eluting, the eluted cells were regarded as CD4^+^CD25^+high^CD127^dim/−^ regulatory cells for further analysis.

### Assessment of Treg purity

CD4FITC/CD25APC (BD Company, USA) and eBioscience Flow Cytometry Staining buffer (eBioscience, USA) were used in two groups (experimental and negative groups). We followed the eBioscience Treg kit procedure in which cells were suspended by vortex shock and an eBioscience Fixation and Permeabilization set added. After a depleting wash and repeated removal of supernatant, cells were reacted with *Foxp*3 antibody. Multiparametric FACS analysis for the detection of the various markers was performed using a FACS Calibur system (BD, USA).

### RNA extraction

Total RNA was extracted from Tregs using Trizol reagent (Invitrogen, Carlsbad, California, USA) and following the protocol for an miRNeasy mini kit (Qiagen, USA). The quantity of the RNA samples was assessed using an ultraviolet spectrophotometer (NanoDrop-1000 Technologies).

### miRNA array analyses

The miRCURY LNA Array (miRBase 18.0) system was performed in three patients with ITP and three healthy donors as previously described.^9^ The RNA samples were then labeled with a miRCURY Array Power labeling kit (Exiqon, Denmark) and hybridized on a miRCURY LNA Array (miRBase 18.0) station and scanned with an Axon GenePix 4000B microarray scanner (Applied Biosystems, USA). The significance of upregulation or downregulation of miRNAs was determined with a fold change of >1.3 and a padj<0.05 calculated by Student’s t-test.

### TaqMan qPCR for quantification of miRNAs

Total RNA was extracted using Trizol reagent (Invitrogen) following the manufacturer’s protocol. Primers (Invitrogen, Shanghai) were designed using Primer 5.0 software (tables 1 and 2). A reverse transcriptional kit (Epicentre, USA) and a TaqMan MicroRNA Assay kit (Superarray, USA) were used for qPCR assays. The manufacturer’s instructions were strictly followed.

**Table 1:** RT primer of u6 and miRNA

Primer name	RT primer
U6	5’CGCTTCACGAATTTGCGTGTCAT3’
miR-155–5p	5’GTCGTATCCAGTGCGTGTCGTGGAGTCGGCAATTGCACTGGATACGACACCCCT3’
miR-146b-5p	5’GTCGTATCCAGTGCGTGTCGTGGAGTCGGCAATTGCACTGGATACGACAGCCTATG3’
miR-142–3p	5’GTCGTATCCAGTGCGTGTCGTGGAGTCGGCAATTGCACTGGATACGACTCCATAAA3’

**Table 2:** PCR primer of u6 and miRNA

Primer name	RT primer
U6	F:5’GCTTCGGCAGCACATATACTAAAAT3’ R:5’CGCTTCACGAATTTGCGTGTCAT3’
miR-155–5p	GSP:5’GGGTTAATGCTAATCGTGA3’ R:5’TGCGTGTCGTGGAGTC3’
miR-146b-5p	GSP:5’GGGGGTGAGAACTGAATT3’ R:5’TGCGTGTCGTGGAGTC3’
miR-142–3p	GSP:5’GGGGGTGTAGTGTTTCCTA3’ R:5’CAGTGCGTGTCGTGGA3’

U6 small nuclear RNA was quantified as a control to normalize differences in total RNA levels.

PCRs were amplified on an ABI Prism-7900 Sequence Detection System (Applied Biosystems, USA). An initial denaturation at 95°C for 10 min was followed by 40 cycles of denaturation at 95°C for 10 s, and extension at 60°C for 1 min. A dissolution curve was drawn to confirm that the PCR specific product was as specified.

### Statistics

Differences in the expression of miRNAs were analyzed using Volcano Plot software, and clustering was analyzed using MEV software (V.4.6, TIGR).

The relative quantity of gene expression was obtained by comparing the relative expression of U6 using a 2^–ΔΔCt^ method (Ct_sample_ – Ct_u6_). A mean±SE error or median were used to describe numerical data. The relative levels of expression of miRNAs were analyzed using a nonparametric Mann–Whitney U test and Spearman correlation analysis. IBM SPSS Statistics for Windows, V.18.0 was used for statistical processing and p<0.05 was considered statistically significant.

### Statistical analysis

Statistical analysis was applied by using SAS V.6.12 (SAS Institute, Cary, North Carolina, USA). The miRNAs array data were analyzed by using Volcano Plot software, and the cluster was analyzed by using MEV software (V.4.6, TIGR). The 2^−ΔCT^ method was used to analyze the relative changes in gene expression from real-time quantitative PCR experiments. A median and IQR was presented to describe data. Correlation analysis of miRNAs were calculated by Spearman rank correlation coefficients. P<0.05 was considered statistically significant.

## Results

### Assessment of Treg purity

**Figure 1:**
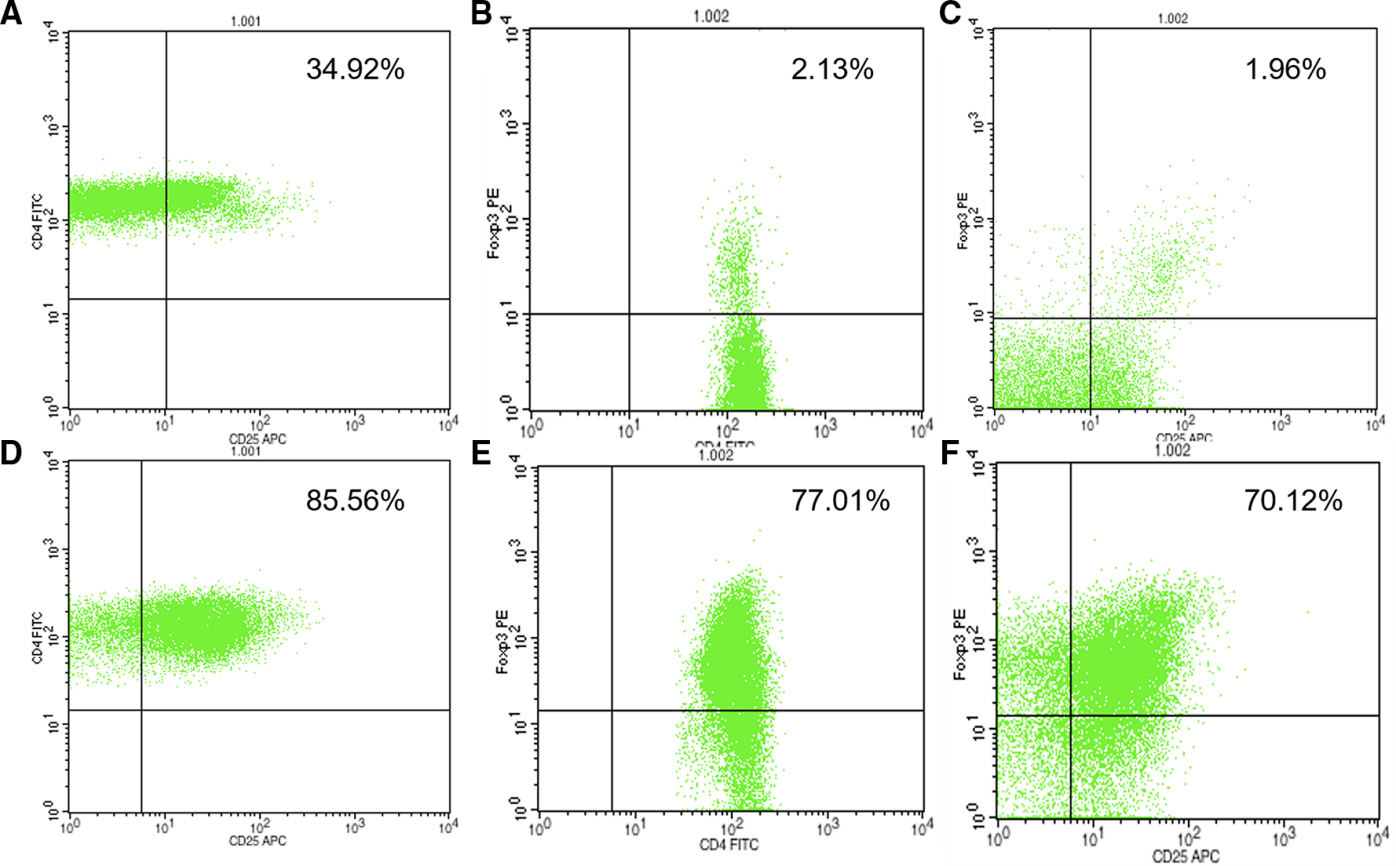
CD4^+^CD25^+^FOXP3^+^Treg cell analysis by FACS: CD4^+^CD25^+^ T cell before sorting (A); CD4^+^FOXP3^+^ T cell before sorting (B); CD4^+^CD25^+^FOXP3^+^ T cell before sorting (C); CD4^+^CD25^+^ T cell after sorting (D); CD4^+^FOXP3^+^ T cell after sorting (E); CD4^+^CD25^+^FOXP3^+^ T cell after sorting (F); CD4^+^FOXP3^+^ Tregs and CD4^+^CD25^+^FOXP3^+^ Tregs were more than 80% and 70%, respectively. FACS, fluorescence activated cell sorter.

Purified Tregs were analyzed for their surface expression of CD4, CD25, *Foxp3* antigens, and the cell counts were about 1–2×10^5^. FACS analysis showed that CD4^+^ *Foxp3* ^+^ Tregs were more than 80% and CD4^+^CD25^+^ *Foxp3* ^+^ Tregs were more than 70% (figure 1).

### Differential expression of miRNAs in Tregs of patients with ITP compared with healthy controls

**Figure 2:**
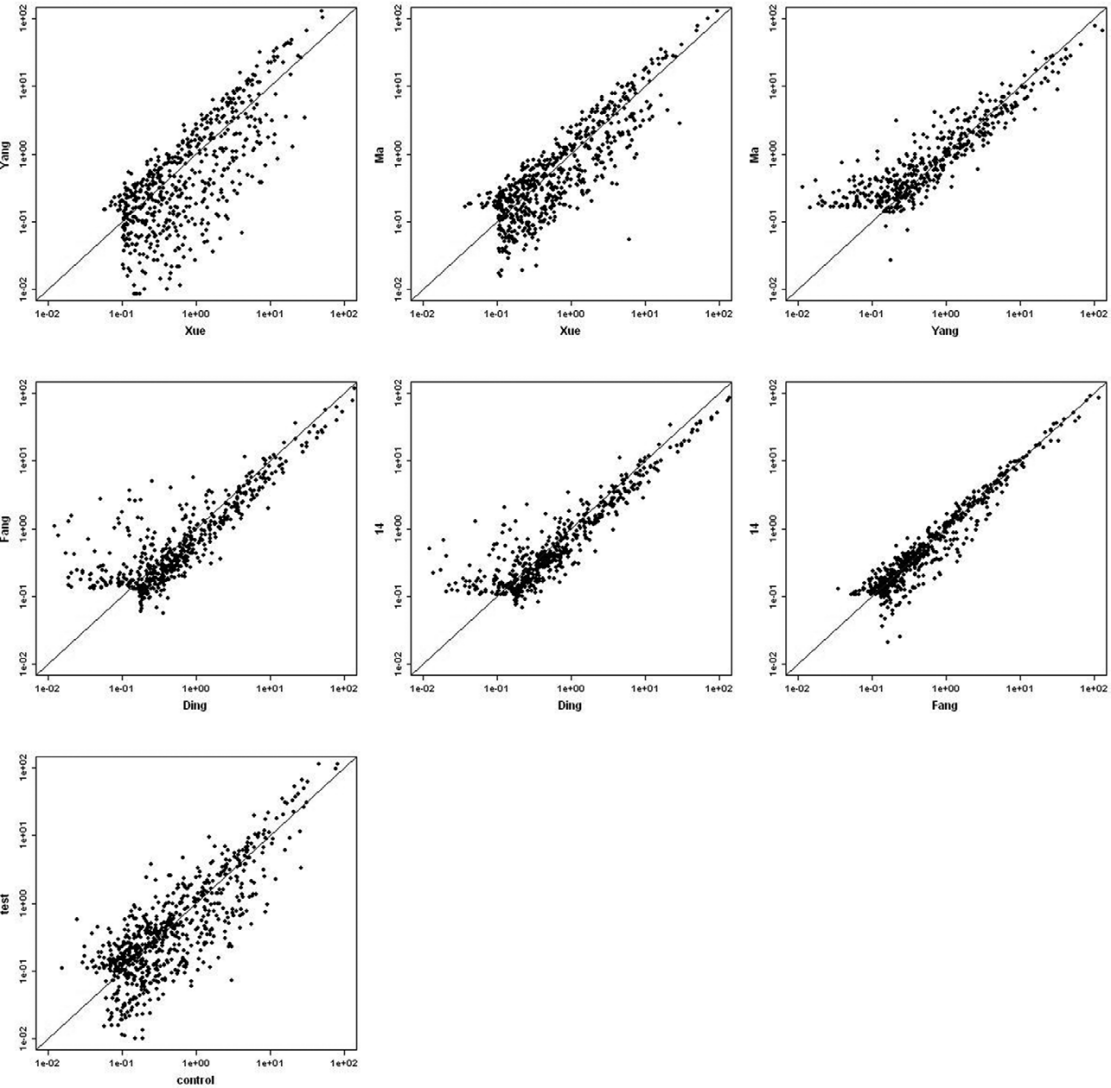
Correlation scatter plot of gene hybridization signal in microarray. The figure shows an increased, but not strong hybridization signal.

We chose qualified RNA for microarray and found no signal in negative controls. Experiment quality and sample properties were assessed by the correlation of gene hybridization signal (figure 2). The figure shows an increased, but not strong hybridization signal. One reason for this outcome may be the quality of miRNAs; other reasons might include the weak expression of some miRNAs in the sample or the presence of as yet unknown genes.

**Figure 3:**
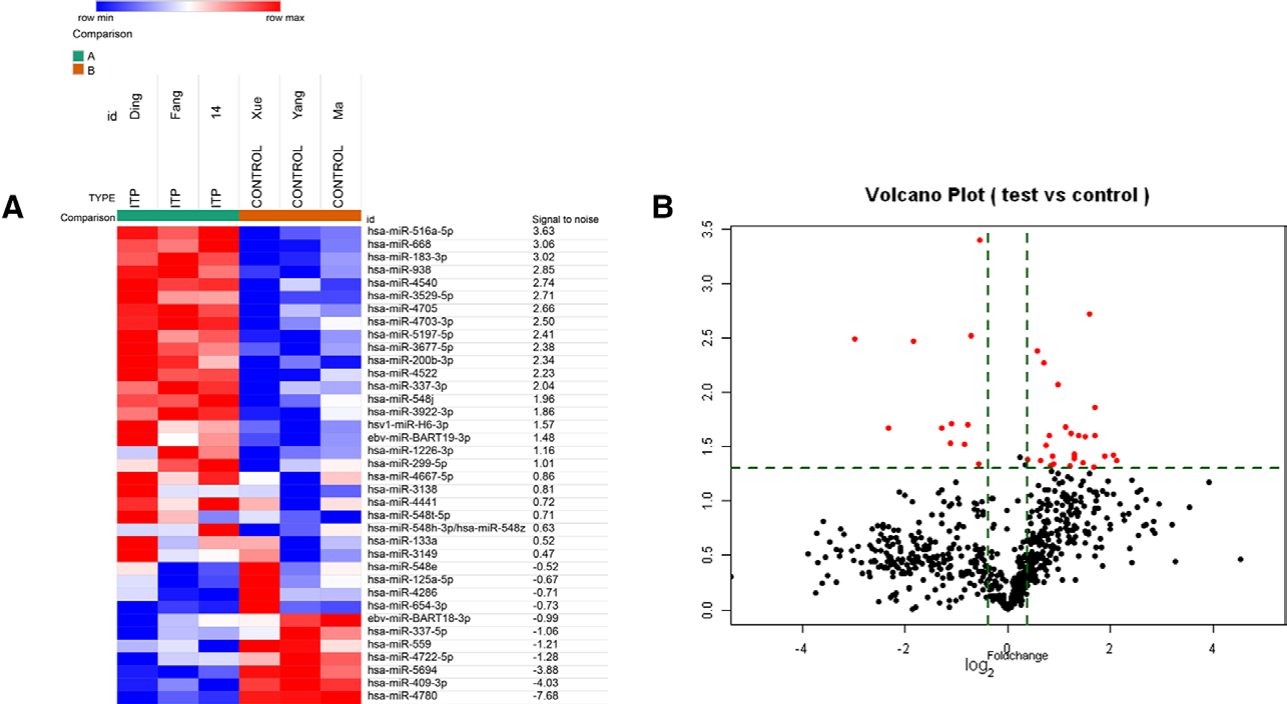
miRNAs clustering analysis by miRCURY LNA Array in three patients with ITP and three healthy donors. Among these, 26 miRNAs were upregulated and 11 were downregulated by a t test using a 1.3-fold change analysis. (A) The heat map of the 37 differentially expressed miRNAs between ITP samples and control samples (>1.3-fold; p<0.05). (B) miRNAs with fold change >1.3 and p<0.05 were plotted in the volcano plot. ITP, immune thrombocytopenia.

**Figure 4:**
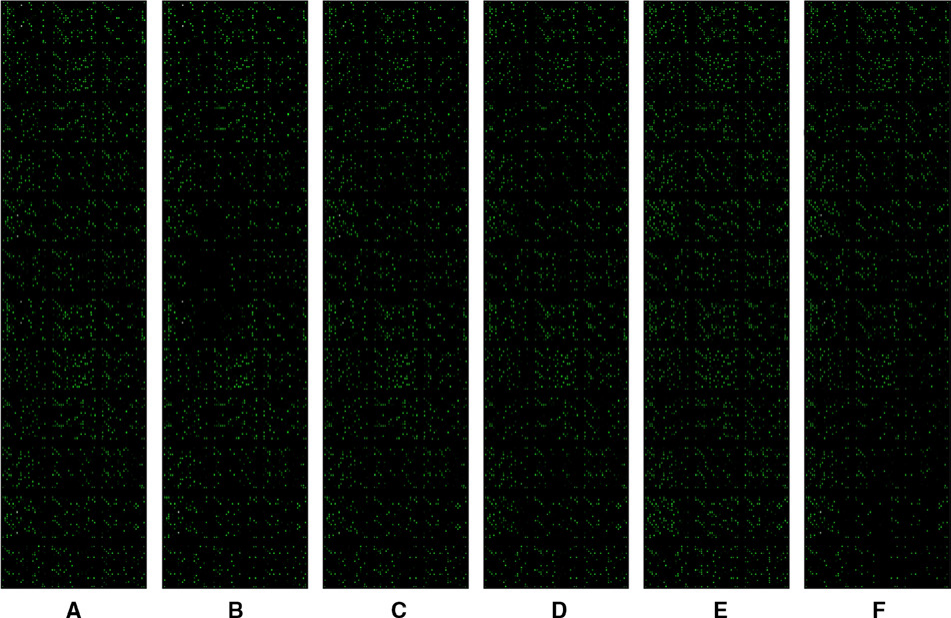
Scanning image of MicroRNA array in Tregs. (A–C) Healthy controls; (D–F) patients with primary immune thrombocytopenia.

We assessed the levels of expression of human miRNAs in Tregs from three patients with ITP and three healthy donors using an miRCURY LNA Array (figures 3 and 4). We found that 502 genes were expressed differentially; 244 had increased expression and 258 decreased expression. Because too few transcripts showed a fold-change >1.3, genes that reached the significance level of p<0.05 and with a fold-change >1.3 were analyzed further. We identified 37 miRNAs differentially expressed in Tregs from patients with ITP compared with healthy controls (tables 3 and 4). Among them, 26 miRNAs were statistically upregulated and 11 were statistically downregulated.

**Table 3:** Upregulated miRNAs by miRNAs array

miRNA	Fold change	P value
hsv1-miR-H6–3p	4.134943	0.037759
hsa-miR-200b-3p	3.214671	0.025018
hsa-miR-668	2.985206	0.001874
hsa-miR-548j	2.359644	0.023893
hsa-miR-3138	2.743772	0.044483
ebv-miR-BART19-3p	3.184072	0.04792
hsa-miR-3677–5p	2.444652	0.036854
hsa-miR-3529–5p	4.319884	0.042622
hsa-miR-133a	1.49291	0.004163
hsa-miR-5197–5p	2.819095	0.025433
hsa-miR-4540	2.454396	0.039986
hsa-miR-4667–5p	1.752593	0.02491
hsa-miR-548h–3p/hsa-miR-548z	1.660791	0.030347
hsa-miR-183–3p	2.171599	0.020913
hsa-miR-4522	2.317606	0.047492
hsa-miR-548t–5p	1.306963	0.040787
hsa-miR-3922–3p	1.762495	0.047063
hsa-miR-4441	1.823415	0.038232
hsa-miR-337–3p	1.962377	0.008488
hsa-miR-516a–5p	3.215864	0.013648
hsa-miR-4703–3p	2.448984	0.038187
hsa-miR-4705	2.582836	0.025136
hsa-miR-938	3.702528	0.038265
hsa-miR-299–5p	1.634267	0.005265
hsa-miR-1226–3p	1.859578	0.045089
hsa-miR-3149	1.557769	0.042242

**Table 4:** Downregulated miRNAs by miRNAs array

miRNA	Fold-change	P value
hsa-miR-409–3p	0.281322	0.003337
hsa-miR-125a–5p	0.6731	0.044941
hsa-miR-548e	0.560181	0.029698
hsa-miR-5694	0.408008	0.021335
hsa-miR-559	0.611151	0.003017
hsa-miR-654–3p	0.198366	0.021035
hsa-miR-4780	0.126252	0.003205
hsa-miR-4286	0.46207	0.029317
hsa-miR-337–5p	0.470205	0.0192
ebv-miR-BART18–3p	0.686011	0.000396
hsa-miR-4722–5p	0.585287	0.019703

**Figure 5:**
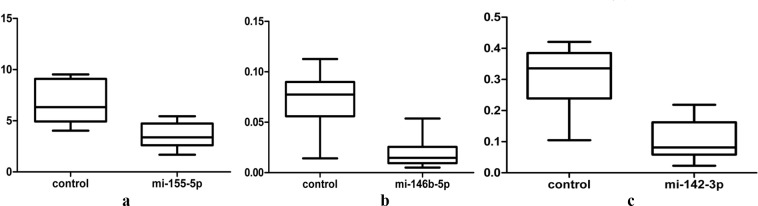
miRNA array results were verified by qPCR inTregs of 18 patients with primary ITP and 15 healthy controls. All miRNAs were abnormally expressed in Tregs of patients with ITP. ITP, immune thrombocytopenia.

Decreased expression level of miR-155–5p, miR-146b-5p, and miR-142–3p in Tregs of patients with ITP miRNA microarray analysis revealed that 37 aberrant miRNAs were identified. We validated the results by TaqMan qPCR in 18 patients with ITP and 15 healthy controls and found three abnormally expressed miRNAs in Tregs from patients with ITP (table 5, figure 5). Then, we compared the level of expression of miRNA in Tregs by correlation analysis and found none were significantly different (table 6).

**Table 5:** Relative expression level of miRNAs

miRNA	Relative expression level		P value
	Healthy control	Patient with ITP	
miR-155–5p	6.633 (4.03–9.53)	3.390 (1.68–5.44)	0.003
miR-146b-5p	0.08 (0.07–0.11)	0.015 (0.01–0.05)	0.001
miR-142–3p	0.36 (0.10–0.42)	0.08 (0.05–0.22)	0.002

**Table 6:** Correlation analysis of miRNAs

miRNA	miR-155–5p		miR-146b-5p		miR-142–3p	
	*r*	P value	r	P value	r	P value
miR-155–5p	–				0.026	0.92
miR-146b-5p	0.013	0.96				
miR-142–3p	–		0.432	0.073		

## Discussion

The expression of CD25 and the Treg-specific *Foxp3* were the characteristics of Treg which is essential for basic function.^10^ Severe inflammation and autoimmunity could be observed in mice and humans with dysfunctional Tregs, indicating that Tregs may help prevent harmful autoimmune responses. Dysregulation of Tregs could also be considered to associate with ITP pathophysiology. A decreased frequency of Tregs in peripheral blood is found in patients with ITP,^4 11^ and their immunosuppressive function was inferior in patients with ITP compared with healthy controls.^12 13^ In the present study, we sorted Tregs from patients with ITP and healthy controls using immunoreactive magnetic beads to study the pathogenesis of ITP and dysfunction in Tregs.

It has become more clear that miRNAs form a complex signaling pathway and aberrant expression of miRNAs is implicated in the pathogenesis of autoimmune diseases.^14 15^ miRNAs also play a part in growth development, maintaining immune function and inhibiting the inflammatory response in Tregs.^16–18^ At present, the association of miRNAs with ITP is poorly understood^19–21^ and there is no report regarding miRNA microarray analysis of Tregs from patients with ITP. Among results of miRNAs tested, we found by clustering analysis that 37 were differentially expressed in Tregs from patients with ITP compared with healthy controls. We concluded that miRNAs contribute to the pathogenesis of ITP by disrupting the function of Tregs. However, the immune regulation by miRNAs is complicated and elucidating the specific mechanism requires further study.

Our previous studies confirmed that Treg cells were involved in the pathogenesis of ITP. We found that the number of Treg cells in peripheral blood of patients with ITP was significantly lower than that of normal control group, and the immunosuppressive function of Treg cells was significantly weakened. In this experiment, although 37 kinds of miRNAs were abnormally expressed, combined with previous literature, it was reported that miR-155–5p, miR-146b-5p, and miR-142–3p may be involved in regulating the development and function of Treg cells. On the other hand, as the limited sample size of patients, we initially selected miR-155–5p, miR-146b-5p, and miR-142–3p in patients with ITP for qPCR verification. We confirmed by qPCR that miR-155–5p, miR-146b-5p, and miR-142–3p were downregulated in Tregs of patients with ITP compared with healthy controls. miR-155 is located in exon 3 of chromosome 21 and is regulated by cytokines expressed by T cells, including IL-10^22^ and TGF-β.^23^ A suppressor of cytokine signaling (SOCS1) and signal transduction activated factor are targets of miR-155.^16 24 25^ Kohlhaas^26^ reported that miR-155-deficient mice have reduced numbers of Tregs, both in the thymus and periphery, because of impaired development and increasing miR-155 level that contributes to enhancing the immunosuppressive function of Tregs. These findings indicate that miR-155 contributes to the function and development of Tregs. We found that miR-155 was significantly downregulated in Tregs, which may lead to Treg dysfunction and contribute to ITP pathogenesis. miR-146 is composed of miR-146a and miR-146b, which locate on chromosomes 5 and 10, and have only two bases different between them. Therefore, it is not surprising that their mechanism of action and target gene are similar.^27^ miR-146 inhibits tumor necrosis factor receptor-related factor 6 and interleukin-1 receptor-associated kinase 1 at a post-transcriptional level through NF-κB signaling pathways and negatively regulates immune signal transduction.^28^ miR-146a contributes to the development of Tregs and to maintaining their immunosuppression function. We found that miR-146a is significantly decreased in peripheral blood cells from patients with ITP. We know that in patients with ITP, T helper Type 1 (Th1) cells are in a polarization mode, and damaging Treg function results in deficiencies of IFNγ, IL-10, and other cytokines. Lu *et al* 29 reported that miR-146a, an miRNA prevalently expressed in Tregs, is critical for their suppressor function. A breakdown of immunological tolerance was led by a deficiency of miR-146a in Treg cells which manifested in fatal IFNγ-dependent immune-mediated lesions in a variety of organs. Augmented expression and activation of signal transduction and activator transcription 1 (Stat1), a direct target of miR-146a may be the crime culprit. Similarly, heightened Stat1 activation in Tregs subjected to a selective ablation of SOCS1, a key negative regulator of Stat1 phosphorylation downstream of the IFN-γ receptor, was associated with analogous Th1-mediated pathology-associated autoimmunity. miR-146b is also expressed abnormally in many tumor diseases.^30–32^ The literature regarding miR-146 and ITP is scant. In the present microarray and PCR experiments, we found that miR-146b was significantly decreased in Tregs from patients with ITP compared with healthy controls and so we postulated that miR-146 is involved in the immunoregulation mechanism of Tregs in ITP. miR-142–3p is located in exon 1 on chromosome 17 and miR-142–3p-dependent functions have been identified in hematopoiesis,^33^ the immune system, and in relation to hemato-oncological diseases.^34^ miR-142–3p also has a certain relationship with Tregs. Huang *et al* 35 found that Tregs exert their suppressor function by transferring cyclic adenosine monophosphate (cAMP) to responder T cells. miR-142–3p regulates the production of cAMP by targeting adenylyl cyclase (AC)9 messenger RNA in Tregs. miR-142–3p limits the level of cAMP in Tregs by inhibiting AC9 production, whereas *Foxp*3 downregulates miR-142–3p to keep the AC9/cAMP pathway active in Tregs. We can therefore conclude that miR-142–3p regulates Tregs activity in the course of ITP. miR-142–3p has been reported in autoimmune diseases such as systemic lupus erythematous, but has not been reported to be associated with ITP. Using microarray and qPCR, we confirmed that miR-142–3p is deregulated in ITP.

In summary, we found decreased miR-155–5p, miR-146b-5p, and miR-142–3p in Tregs from patients with ITP. Our data indicate that the abnormal expression of miRNAs in Tregs might play a role in the pathogenesis of ITP. However, an individual miRNA may target multiple genes. Therefore, the function of miRNA in ITP could be far more complex. The mechanisms of aberrant miRNA expression on specific immune cell subsets in ITP warrant further study.
